# Development of a Convenient and Quantitative Method for Evaluating Photosensitizing Activity Using Thiazolyl Blue Formazan Dye

**DOI:** 10.3390/molecules29112471

**Published:** 2024-05-24

**Authors:** Smee Kang, Yeong Ji Oh, Mi-Ri Kim, Yu Na Jung, Eiseul Song, Hyowon Lee, Jungil Hong

**Affiliations:** 1 Department of Food Science and Technology, College of Science and Convergence Technology, Seoul Women’s University, 621, Hwarangro, Nowon-gu, Seoul 01797, Republic of Korea; michael1224@swu.ac.kr (S.K.); k0121i@naver.com (M.-R.K.); jyn5537@swu.ac.kr (Y.N.J.); dew2584@naver.com (E.S.); hyowon24@korea.ac.kr (H.L.); 2 Major in Food Science & Biotechnology, Institute of Bio Engineering, College of Future Convergence, Eulji University, Seongnam 13135, Republic of Korea; anabeda@eulji.ac.kr

**Keywords:** MTT, MTT formazan, photosensitizer, photosensitizing activity, quantitative analysis

## Abstract

Photosensitizers cause oxidative damages in various biological systems under light. In this study, the method for analyzing photosensitizing activity of various dietary and medicinal sources was developed using 1-(4,5-dimethylthiazol-2-yl)-3,5-diphenylformazan (thiazolyl blue formazan; MTT-F) as a probe. Significant and quantitative decolorization of MTT-F was observed in the presence of photosensitizers used in this study under light but not under dark conditions. The decolorization of MTT-F occurred irradiation time-, light intensity-, and photosensitizer concentration-dependently. The decolorized MTT-F was reversibly reduced by living cells; the LC-MS/MS results indicated the formation of oxidized products with −1 *m*/*z* of base peak from MTT-F, suggesting that MTT-F decolorized by photosensitizers was its corresponding tetrazolium. The present results indicate that MTT-F is a reliable probe for the quantitative analysis of photosensitizing activities, and the MTT-F-based method can be an useful tool for screening and evaluating photosensitizing properties of various compounds used in many industrial purposes.

## 1. Introduction

Formazan is a dye compound produced by the reduction of tetrazolium salt, and different-colored formazan, including red, blue, and orange, is produced depending on the type of tetrazolium [1,2]. Tetrazolium is a heterocyclic compound bearing three connected aromatic rings, and 3-(4,5-dimethyl-2-thiazolyl)-2,5-diphenyl-2H-tetrazolium bromide (MTT) has been widely used in biological assays for cell viability [3,4]. MTT is reduced by mitochondrial reductases and dehydrogenases in various living cells [5,6,7] to form its corresponding formazan, 1-(4,5-dimethylthiazol-2-yl)-3,5-diphenylformazan (thiazolyl blue formazan; MTT-F), which is insoluble in water and dissolved in organic solvents such as dimethyl sulfoxide (DMSO), ethanol, or in an aqueous solvent containing sodium dodecyl sulfate (SDS) [6,8,9].

The interference of various light sources with formazan produced in cell culture systems depending on the compounds being treated has been reported [10,11,12]. Park et al. [13] reported that MTT-F produced by cells treated with zinc protoporphyrin (ZnPP) was discolored under light, which might cause errors in experiments measuring cell viability. A subsequent study also showed that MTT-F decolorization under light was caused by various porphyrin compounds with photosensitizing properties [10].

Photosensitizing properties have important implications in many areas including food, medicine, and the environment. In particular, photosensitizing compounds in food have been reported to cause spoilage and deterioration of nutrients through activation by light [14]. The photoreactive compounds including natural pigments with a porphyrin structure, such as chlorophyll in vegetables, myoglobin in meat, and riboflavin (RF) in various dietary sources generate ROS through either type I or type II reactions when exposed to light [14]. Although there are a few different opinions, in type I reactions, light-activated photosensitizers act directly on an adjacent substrate to form substrate radicals via electron transfer, triggering a subsequent oxidation chain reaction. In the type II mechanism, photosensitizers exposed to light first react with atmospheric triple oxygen to produce singlet oxygen through energy transfer and then generate different reactive oxygen species (ROS) through a chain reaction [15,16,17]. Singlet oxygen generated by photoreactive compounds in food reacts with unsaturated fatty acids thousands of times faster than normal triplet oxygen, forming oxidation products and destroying nutrients such as vitamins and amino acids [18]. Photosensitizers in foods cause a variety of degradation events, including lipid oxidation by photosensitizing contaminants [19,20], dispersion of milk lipids by RF [14,21], and deterioration of the sensory properties of meat products [22,23]. The activation of photosensitizing compounds by various light sources installed in markets or on commercial shelves is also expected to affect the quality and sensory properties of various food products [24,25]. Accordingly, the detection of photosensitizers and measurement of their activity can be an important approach to predict and control the degradation of food quality caused by them.

On the other side, photosensitizers have a wide range of useful applications in the fields of medicine and the environment. Photodynamic therapy, which uses photosensitizers in combination with appropriate light, has been widely used to treat cancer, periodontitis, and skin diseases [26,27]. In the environmental industry, research has been conducted on pollutant removal using photocatalysis. The catalytic activity of light-activated photosensitizers has also been applied for the removal of volatile substances that cause air pollution [28]. Therefore, in order to discover new photosensitizers that can be used in various fields, it is necessary to develop a convenient method to measure the photosensitizing activity of various natural sources. However, there is very little research that has been conducted on the measurement of this activity.

The present study aimed to develop a quantitative method for analyzing photosensitizing activity using MTT-F dye based on its decolorization properties when exposed to light in the presence of photosensitizers. The assay method presented in this study is expected to provide a convenient and quantitative evaluation of photosensitizing activity, overcoming the drawbacks and limitations of existing methods used to date. In addition, this method is expected to contribute to the understanding of the actions and mechanisms of photosensitizing compounds, as well as to the screening and discovery of photosensitizers that can be used in various academic and industrial fields.

## 2. Results and Discussion

### 2.1. Decolorization of MTT-F by Photosensitizing Agents under Light

**Figure 1:**
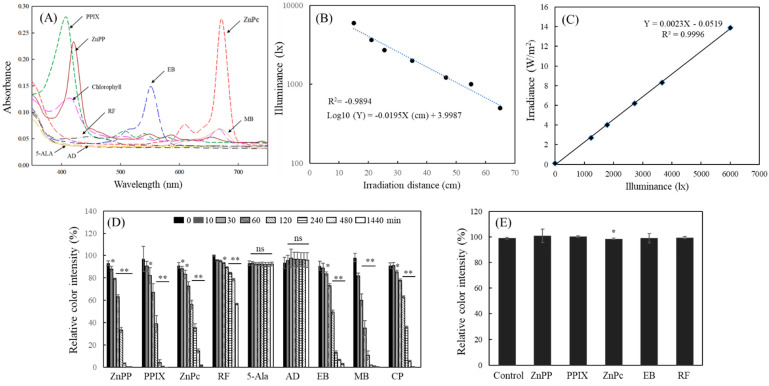
Absorbance spectra of photosensitizers, the fluorescent light characteristics used in this study, and changes in color stability of MTT-F by photosensitizers. Absorbance spectra of photosensitizers including ZnPP, PPIX, ZnPc, RF, 5-ALA, AD, EB, MB (each 5 µM), and CP (50 µg/mL) dissolved in DMSO were analyzed (**A**). A correlation between irradiation distance (cm) and illuminance (lx) (**B**) and between light energy and illuminance (**C**) of the fluorescent light used in this study were shown. MTT-F containing ZnPP, PPIX, ZnPc, RF, 5-ALA, AD, EB, MB (each 5 µM), or CP (50 µg/mL) were also incubated under the fluorescent light (2000 lx) (**D**) or a dark place (**E**). Color intensity of MTT-F was analyzed at 550 nm at different time points during 24 h (**D**) or at 24 h under the dark (**E**). Each value (mean ± SD) represents relative color intensity based on each 0 time control (*n* = 4). *, **; significantly different from control according to Student’s *t*-test (*, *p* < 0.05; **, *p* < 0.01). ns, not significant.

The widely used photosensitizing agents and their precursors, including zinc protoporphyrin (ZnPP), protoporphyrin IX (PPIX), zinc phthalocyanine (ZnPc), riboflavin (RF), 5-aminolevulinic acid (5-ALA), acridine (AD), erythrosine B (EB), methylene blue (MB), and chlorophyll (CP), were used in this study. A general characteristic of photosensitizers is that they absorb light and transfer their energy to the surrounding environment; thus, they have a typical wavelength range for light absorption [29,30]. The light absorption properties of the different photosensitizers used in this experiment were analyzed by dissolving them in dimethyl sulfoxide (DMSO) (Figure 1A). ZnPP, PPIX, and CP exhibited absorption peaks in the short-wavelength range of 400–450 nm (violet-blue) under visible light, indicating a unique absorption region conferred by the porphyrin ring structure shared by these compounds (Figure 1A). EB exhibited a light absorption peak in the red wavelength range near 550 nm. RF also showed an absorption peak at 400–450 nm but had a relatively weak absorption property at the same concentration compared to porphyrin-containing compounds (ZnPP, PPIX, CP). Both ZnPc and MB exhibited a distinct absorption pattern in the long-wavelength visible light range of 650–700 nm. ZnPc, a synthetic photosensitizer, possesses a phthalocyanine structure that enables it to absorb long-wavelength light with excellent penetration into the body, rendering it widely used in photodynamic therapy [31,32]. However, 5-ALA and acridine did not exhibit any clear light absorption peaks at a concentration of 5 µM. The fluorescent light used in this study exhibited emission peaks in at least three regions, 440, 550, and 620 nm, and was therefore considered to be an applicable light source for all photosensitizers used in this study (Figure S1A). The illuminance of the light was inversely correlated with irradiation distance. This fluorescent light produced an illuminance of ~2000 lx at an irradiation distance of 35 cm (Figure 1B), and the irradiance under this condition was ~4 W/m^2^ (Figure 1C). In addition, most photosensitizers did not show any severe interference with MTT-F, with an absorbance peak in the 550 nm region at a concentration of 5 μM, except for EB.

The stability of MTT-F under the irradiation condition (2000 lx) was investigated for 24 h, and no significant color degradation of MTT-F was observed (Figure S1B). However, under the same irradiation conditions, seven compounds, with the exception of 5-ALA and acridine, induced significant MTT-F decolorization (Figure 1D). The photosensitizers with a porphyrin ring structure, such as ZnPP and PPIX, were the most effective for MTT-F decolorization, showing significant changes even after 10 min (Figure 1D). The decolorization reaction of MTT-F by RF, a representative photosensitizer in food [33], was markedly delayed compared that with of other photosensitizers. The photosensitizers that induced rapid formazan decolorization under light irradiation did not affect the color intensity of MTT-F in a dark condition; no marked color change in MTT-F was observed over 24 h (Figure 1E). The decolorization of MTT-F by these compounds occurred only under light irradiation and might therefore be attributed to their reactivity with light, i.e., the expression of photosensitizing activity.

The decolorization of MTT-F occurred by reactions with every structure of the photosensitizers responsible for their photosensitizing properties, including the porphyrin structure of ZnPP, PPIX, phthalocyanine structure of ZnPc, fluorone of EB, phenothiazine of MB, and isoalloxazine of RF. These results suggest that the decolorization of MTT-F by photosensitizers under light exposure is not limited to specific structures, but is a general reaction to substances exhibiting photosensitizing properties. Therefore, MTT-F can be used as a universal probe for the detection and measurement of photosensitive activity by exhibiting the decolorization reaction by photosensitizers in a light exposure time-dependent manner.

### 2.2. Concentration-Dependent Effects of Photosensitizers on MTT-F Decolorization

**Figure 2:**
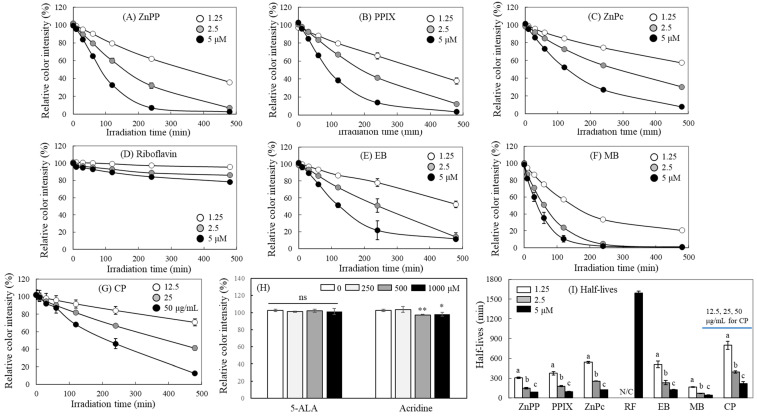
Concentration-dependent effects of different photosensitizers on decolorization of MTT-F. MTT-F (200 µM) mixed with different concentrations of ZnPP (**A**), PPIX (**B**), ZnPc (**C**), RF (**D**), EB (**E**), MB (**F**) (1,25, 2.5, and 5 µM each), CP (12.5, 25, and 50 µg/mL) (**G**), or 5-ALA and acridine (250, 500, and 1000 µM) (**H**) was incubated under the fluorescent light (2000 lx) for 480 min. Half-lives that induce 50% color degradation of MTT-F by each photosensitizer were also calculated (**I**). Each value represents the mean ± SD (*n* = 4). *, **; significantly different from control according to Student’s *t*-test (*, *p* < 0.05; **, *p* < 0.01). N/C; not calculated. Different letters indicate a significant difference (*p* < 0.05) based on one-way ANOVA and Tukey’s HSD test (in (**I**)).

The decolorization response of MTT-F was analyzed under light irradiation by applying different concentrations of each photosensitizer. The decolorization of MTT-F showed a clear concentration dependence on the photosensitizers used (Figure 2). ZnPP, PPIX, ZnPc, EB, and MB induced a significant MTT-F decolorization reaction even at a concentration of 1.25 µM (Figure 2A–C,E,F). In the case of RF, no obvious MTT-F decolorizing effect was observed at low concentrations of 1.25 and 2.5 μM, but a significant difference was noted at 5 μM (Figure 2D). When its concentrations increased to higher than 10 μM, a distinct time- and concentration-dependent MTT-F decolorization pattern was observed.

Choi et al. [10] reported that porphyrin derivatives, such as ZnPP and PPIX, induced severe decolorization of MTT-F produced by cells even at the level of 100–200 nM, causing serious errors in measuring cell viability. Therefore, it can be inferred that MTT-F is a sensitive probe that can induce colorimetric response even at nanomolar levels of specific photosensitizers. We also observed a distinct concentration-dependent pattern in MTT-F decolorization by food additive grade chlorophyll mixtures (CP) containing corn oil (Figure 2G); it is believed that MTT-F can be utilized to analyze the photosensitizing properties of mixtures or extracts from various natural sources, as well as pure ingredients.

Both 5-ALA and acridine, however, failed to induce the MTT-F decolorization even at a 1 mM level (Figure 2H). 5-ALA is known to be a photosensitizer precursor without showing direct photosensitizing activity. It is converted into several types of photoreactive compounds, including ferrous-protoporphyrin, ZnPP, and PPIX [34]. These properties of 5-ALA have been applied to photodynamic therapy. Studies have reported that it attacks cancer cells via ROS generation under light irradiation [35]. Acridine exhibits maximum light absorption properties in the UV region; it does not show any absorption peaks in the visible light region of 400–700 nm (Figure 1A). Accordingly, the fluorescent light used in this study with emission spectrum in the visible region (Figure S1A) could not trigger the photosensitization of acridine, which explains why acridine did not show a clear formazan decolorization effect.

Figure 2I shows the half-lives of MTT-F color degradation caused by individual photosensitizers. A significant decrease in half-life with increasing concentration of each photosensitizer was observed, except for RF, which showed no obvious decolorizing effects below 2.5 μM. The decolorization response of MTT-F on the concentration of most photosensitizers was quantitative; this characteristic can be utilized to compare and quantify the photosensitizing activity of different compounds.

### 2.3. Illuminance-Dependent Effect on MTT-F Decolorization by Photosensitizing Agents

**Figure 3:**
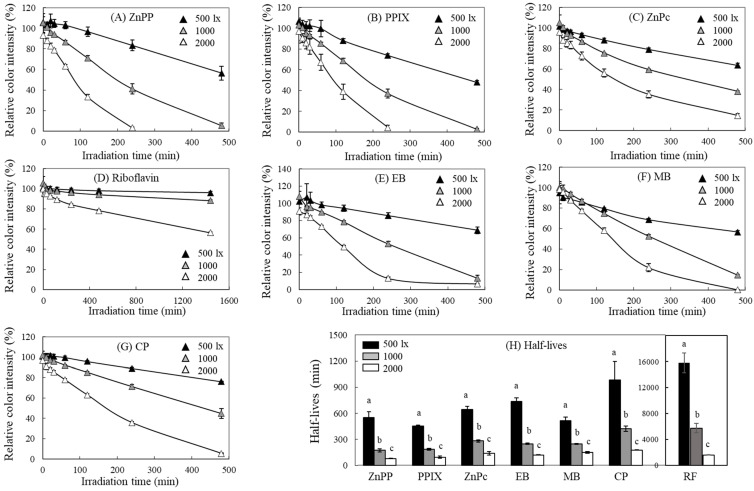
Decolorization of MTT-F by photosensitizers under different fluorescent light intensity. MTT-F (200 µM) mixed with ZnPP (**A**), PPIX (**B**), ZnPc (**C**), RF (**D**), EB (**E**), MB (**F**) (each 5 µM), or CP (50 µg/mL) (**G**) was incubated under different intensities of the fluorescent light (500, 1000, and 2000 lx). At each time point, color degradation of MTT-F was analyzed at 550 nm. Half-lives that induce 50% color degradation of MTT-F by each photosensitizer under different light intensity were also calculated (**H**). Each value represents the mean ± SD (*n* = 4). Different letters indicate a significant difference (*p* < 0.05) based on one-way ANOVA and Tukey’s HSD test in (**H**).

The compounds that induced decolorization of MTT-F in the previous experiment were irradiated with different light intensities to evaluate changes in the extent of MTT-F decolorization. As the intensity of the exposed light increased, all of the photosensitizers used in the experiment caused a much faster decolorization of MTT-F (Figure 3). Porphyrin compounds including ZnPP and PPIX, which showed strong activity in Figure 2, induced a ~50% of MTT-F decolorization within 8 h even at 500 lx (Figure 3A,B). Comparing the period required for 50% decolorization of MTT-F by photosensitizers, there was a significant change in half-lives under different illuminance (Figure 3H). These results indicate that the degree of MTT-F decolorization is proportional to the intensity of the exposed light with stronger photosensitizing activity being expressed under stronger light. Since most photosensitizers induced significant MTT-F decolorization even at light intensities as low as 500 lx, MTT-F is thought to be a sensitive probe for measuring photosensitizing activity.

The activity of currently used photosensitizers was compared using a fluorescent probe, 2′,7′-dichlorofluorescein (DCFH), to confirm that the MTT-F decolorization was due to their photosensitizing activity. DCFH is frequently used for measuring ROS, since a non-fluorescent DCFH is oxidized by ROS (H_2_O_2_, ·OH, ROO· etc.) or RNS (NO·, NOO·, etc.) and converted to highly fluorescent DCF [36,37]. This method was based on the principle that light irradiation of photosensitizers generates ROS, which can convert DCFH into fluorescent DCF. The results indicated that the fluorescent intensity enhanced by each photosensitizer under the same irradiation condition using a fluorescent light (2000 lx) was generally proportional to its concentration and irradiation time (Figure S2). However, 5-ALA and acridine did not induce a significant increase in DCF fluorescence compared to the control, which was consistent with the results using the MTT-F probe (Figure S2C). The increase in DCF fluorescence by photosensitizers did not occur without light irradiation (Figure S2D).

As another approach to validate the current method using MTT-F, the levels of lipid peroxidation products formed by the activation of the photosensitizers used in this experiment were compared (Figure S3). Thiobarbituric acid (TBA) reacts with malondialdehyde produced during lipid peroxidation to form red TBA-reactive substances (TBARS), which can be colorimetrically quantified to determine the extent of lipid oxidation [38,39]. Most of the photosensitizers that induced decolorization of MTT-F also caused evident lipid peroxidation in this method, significantly increasing TBARS levels. However, the potency was somewhat different from what was observed in the method using MTT-F. In particular, the photosensitizers of the porphyrin family including ZnPP, PPIX, and ZnPc showed relatively weak activity (Figure S3A); it is likely that the hydrophobic photosensitizers did not function properly in the current oil-in-water lipid peroxidation system. In this method, RF did not show concentration-dependent activity; the lipid peroxide produced rather decreased at higher RF concentrations (Figure S3B). Acridine and 5-ALA did not show photosensitizing activity at all concentrations (Figure S3C). These three assay methods using MTT-F, DCFH, and TBA showed relatively consistent results for the expression of their activity, although there were some differences in the potency of photosensitizing activity of each compound. Therefore, these results can provide evidence for the validity of the MTT-F method developed in this study for measuring photosensitizing activity.

### 2.4. Decolorization Pattern of MTT-F under Various Conditions

Typically, photosensitizers express their photosensitizing activity through two pathways, including type I or type II reactions. In type I reactions, electron transfer occurs between the activated photosensitizer and an adjacent substrate. In type II reactions, photosensitizers first activate surrounding oxygen molecules by transferring energy to produce singlet oxygen [15,16,17]. The results derived in Figures S2 and S3 are based on the measurement of ROS or lipid oxidation products generated through the expression of photosensitizing activity, rather than the assessment based on the direct reaction of a probe with activated photosensitizers. In the subsequent experiment, it was investigated whether photosensitizer-induced decolorization of MTT-F occurs via the direct and type I-like reaction between the activated photosensitizer and MTT-F via transferring electron. It was hypothesized that certain non-oxygen radical molecules could mimic light-activated photosensitizers and react directly with MTT-F as their adjacent substrate before reacting with oxygen molecules. Accordingly, relatively large and stable radicals capable of direct electron transfer reactions with MTT-F such as (2,2-diphenyl-1-picrylhydrazyl) (DPPH), [2,2′-azino-bis(3-ethylbenzothiazoline-6-sulfonic acid)] (ABTS), and [2,2′-azobis(2-methylpropionamidine) dihydrochloride] (AAPH), were selected as molecules to simulate a reaction with light-activated photosensitizers.

**Figure 4:**
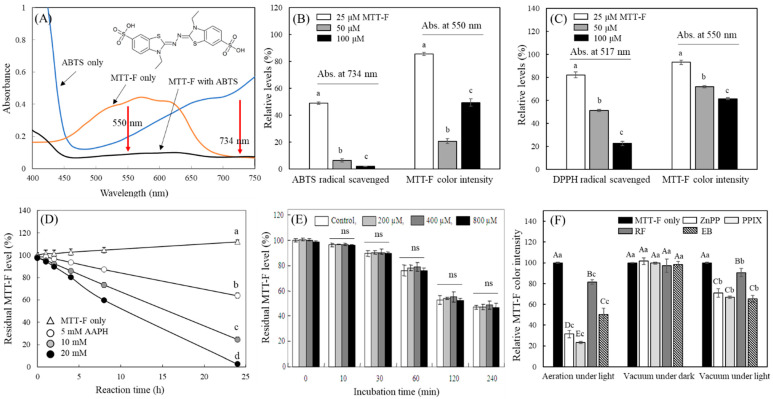
Reaction properties of MTT-F with different radicals or with photosensitizers under vacuum condition. Different concentrations of MTT-F were incubated with ABTS (**A**,**B**) and DPPH (**C**) radicals in the dark. Changes in absorbance spectra (**A**) and the peak absorbances of MTT-F and ABTS (**B**) or DPPH (**C**) were analyzed. MTT-F was incubated with different concentrations of AAPH radical for 24 h (**D**) and H_2_O_2_ for 240 min (**E**). MTT-F was also incubated in the presence of ZnPP, PPIX, EB (25 μM each), or RF (50 μM) under normal or vacuum (~650 mmHg) conditions in the dark or with irradiation of a fluorescent light for 15 min (**F**). Each value represents the mean ± SD (*n* = 4–12). Different letters indicate a significant difference (*p* < 0.05) (**B**–**D**), and different upper and lowercase letters indicate a significant difference among groups under the same conditions and groups treated with the same photosensitizers, respectively, (**F**) based on one-way ANOVA and Tukey’s HSD test. ns; not significant.

The reaction of MTT-F with ABTS radical resulted in a marked decrease in absorbance at 550 nm, the peak absorbance region of MTT-F and a concomitant decrease in absorbance at 730 nm, the peak region of ABTS radical (Figure 4A). The decrease in absorbance of the ABTS radical at 730 nm was dependent on the concentration of MTT-F and occurred immediately after the reaction between MTT-F and ABTS radical (Figure 4B). The decolorization of MTT-F (25 and 50 μM) at 550 nm by ABTS radical occurred sharply. At 100 μM, the intensity of MTT-F increased rather than at 50 μM, which was likely due to excess MTT-F remaining after reaction with ABTS radical (Figure 4B). The effects of DPPH radicals on MTT-F decolorization were also evaluated. A decrease in absorbance at 517 nm, indicating a reduction of the DPPH radical, and MTT-F decolorization at 550 nm were also observed simultaneously; the reduction of DPPH radical and decolorization occurred in a MTT-F concentration-dependent manner (Figure 4C). The decolorization of MTT-F was also consistently observed by reaction with AAPH radical, demonstrating a concentration dependence on the radical (Figure 4D). These results indicate that MTT-F decolorization occurs through a direct reaction with specific non-oxygen radicals, including ABTS, DPPH, as well as AAPH, and that these radicals are also scavenged by electrons donated by MTT-F concomitantly. Accordingly, it is predicted that MTT-F decolorization by photosensitizing agents appears through the type I reaction between MTT-F and activated photosensitizers. Since the MTT-F decolorization by these activated photosensitizer-like radicals occurs in the dark, light-independent interference by these substances in the measurement of photosensitizing activity can be excluded by comparing the results with those under light irradiation. The treatment of MTT-F with H_2_O_2_ resulted in the decolorization of MTT-F over time, but no H_2_O_2_ concentration-dependent decolorization of MTT-F was observed. Even 800 μM H_2_O_2_ did not accelerate the decolorization of MTT-F (Figure 4E). H_2_O_2_, a type of ROS, is a potent oxidizing agent and can be converted to different types of ROS [40]. The present results indicate that H_2_O_2_ did not affect MTT-F decolorization at all, except that a decrease in color was induced by the instability of MTT-F in the aqueous phase. Thus, the MTT-F decolorization appears to be due to a direct reaction with a light-activated photosensitizer and not the result of a reaction with ROS generated by photosensitizers.

The decolorization of MTT-F by different photosensitizers was also observed even under vacuum conditions, albeit to a lesser extent (Figure 4F), indicating that the reaction could also occur independently of the presence of atmospheric oxygen or other gaseous molecules. These results suggest that the MTT-F decolorization is preferentially caused by the type I-like reaction that reacts directly with the activated photosensitizer rather than the type II reaction driven by ROS generated by the photosensitizer. Therefore, unlike indirect methods analyzing ROS or lipid oxidation products (TBARS) produced by photosensitizing reactions, the current MTT-F decolorization method could be an innovative method for analyzing the photosensitizing activity caused by the direct reaction of MTT-F probe with light-activated photosensitizers.

### 2.5. Determination of MTT-F Product Decolorized by Photosensitizers

**Figure 5:**
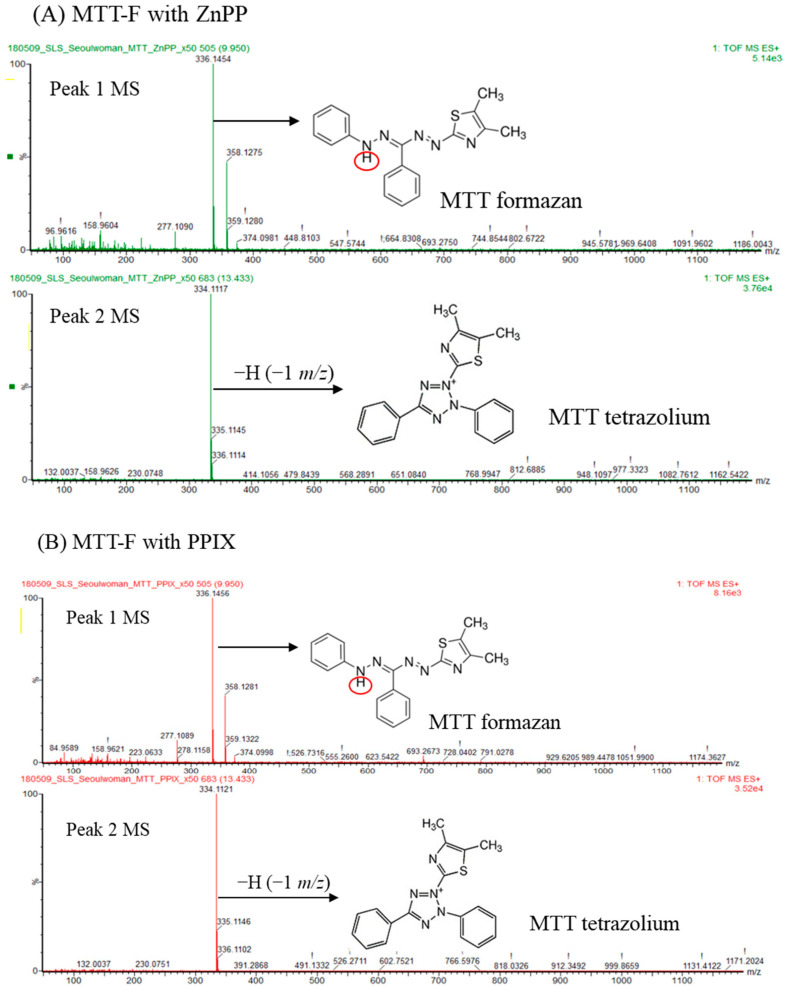

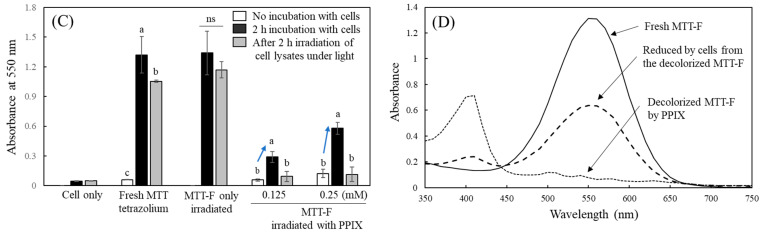
Identification and characterization of decolorized MTT-F by photosensitizers and its reduction by living cells. The MS spectra of the reaction products from MTT-F incubated with ZnPP (**A**) and PPIX (**B**) under light were shown as described in the materials and method. MTT-F (2.5 mM) was decolorized after incubation of PPIX (50 µM) under light, and the decolorized MTT-F diluted 10 times with culture media, fresh MTT tetrazolium, or MTT-F irradiated without PPIX were incubated with HaCaT cells for 2 h. MTT-F color intensity was analyzed at 550 nm after cell lysed immediately and 2 h incubation under a fluorescent light (**C**). Absorbance spectra of fresh MTT-F, MTT-F decolorized by PPIX, and a cell-reduced MTT-F were also compared (**D**). Different letters indicate a significant difference (*p* < 0.05) based on one-way ANOVA and Tukey’s HSD test (**C**). ns; Not significant.

The oxidation products obtained from the reaction of MTT-F with ZnPP or PPIX were analyzed using a UPLC-ESI-MS system to identify the decolorized products of MTT-F by photosensitizers (Figure 5). Two peaks with retention time of ~10 (peak 1) and ~14 (peak 2) min were obtained by the current UPLC from the half-decolorized products after reacting MTT-F with ZnPP (Figure S4A). An MS analysis of peak 1 revealed the base peak at *m*/*z* ~336 in ESI+ mode, which was expected to be non-oxidized MTT-F (C_18_H_17_N_5_S, FW 335.4). An MS analysis of peak 2 showed the base peak at *m*/*z* 334~335 as major products. These were expected to be an oxidized product in which one hydrogen atom was abstracted from the parent MTT-F molecule (Figure 5A). The peaks with the similar retention times were also observed in the reaction mixture of MTT-F and PPIX (Figure S4B). The mass spectra from peaks 1 and 2 showed a similar pattern to the reaction of MTT-F with ZnPP, with *m*/*z* ~336 and *m*/*z* 334~335 as the base peaks, respectively (Figure 5B). These results suggest that MTT tetrazolium (C_18_H_16_N_5_S, FW 334.4) is the major decolorized product that has lost a hydrogen atom (−1 *m*/*z*) from MTT-F. A further MS/MS analysis of peak 2 showed that the distinct fragment pattern at *m*/*z* 306, 231, 230, 203, 127, and 77 was commonly detected in the oxidation products of MTT-F by ZnPP and PPIX indicating a common oxidation pathway and reaction products of MTT-F occur regardless of the photosensitizer (Figure S4C,D).

If MTT-F is converted to its corresponding tetrazolium by light-activated photosensitizers, the decolorized products can also be reversibly reduced to MTT-F by living cells. In the following experiments, MTT-F decolorized by PPIX was treated with living HaCaT cells for 2 h, and its cell-mediated reduction was evaluated (Figure 5C). As a result, the color intensity at 550 nm of MTT-F decolorized by PPIX was significantly enhanced by the treated cells, similarly to the case with fresh MTT tetrazolium treatment. Interestingly, the absorbance at 550 nm of the lysates containing intracellularly reduced MTT-F decreased again after 2 h of irradiation, possibly due to the influence of residual PPIX co-treated with decolorized MTT-F (Figure 5C). The absorbance spectrum of the MTT-F decolorized by PPIX and recovered by cells closely matched that of fresh MTT-F (Figure 5D). Based on these observations, it is believed that the major decolorized product of MTT-F by photosensitizers is MTT tetrazolium; it can be reversibly reduced by cells and then oxidized by photosensitizers again. It is also necessary to examine in more detail the oxidation pathway and reaction products of MTT-F by different photosensitizers.

### 2.6. Quantification of Photosensitizing Activity Using MTT-F

**Figure 6:**
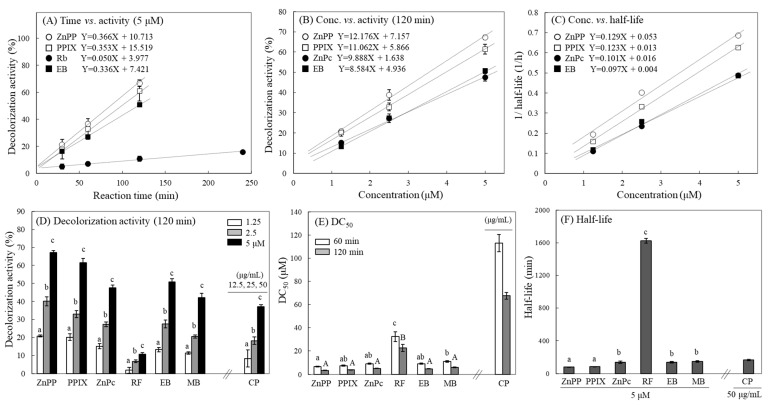
Correlation between photosensitizing activity and various reaction factors, and comparison of different calculation protocols for photosensitizer activity. Photosensitizing activities of ZnPP, PPIX, EB, and Rb or ZnPc as representatives were correlated with the reaction factors including irradiation time (**A**), concentration of the photosensitizer applied (**B**), and formazan half-life (**C**). Photosensitizing activities of different photosensitizers were also calculated based on MTT-F decolorization activities at 120 min (**D**), DC_50_ (concentration that requires 50% decolorization of MTT-F at 60 and 120 min) (**E**), and half-lives (time that requires 50% decolorization of MTT-F by 5 µM or 50 µg/mL of each photosensitizer) (**F**). Each value represents the mean ± SD (*n* = 4). Different letters indicate a significant difference (*p* < 0.05) among different concentrations (**D**) or different photosensitizers (**E**,**F**) based on one-way ANOVA and Tukey’s HSD test.

According to our results, the degree of MTT-F decolorization by photosensitizers has been shown to increase quantitatively with the irradiation time, light intensity, and concentration of photosensitizers. Therefore, it was expected that the activity of various photosensitizers could be quantified and compared based on these reaction characteristics. Thus, a correlation between these reaction parameters including reaction (irradiation) time and photosensitizer concentration and the photosensitizing activity based on MTT-F decolorization was investigated (Table 1). First, the degree of MTT-F decolorization induced by each photosensitizer was converted into decolorization activity (DA). To determine the correlation between reaction time and DA, the irradiation time range was set to 0–240 or 480 min, where the decolorization activity of MTT-F remained linear, and each photosensitizer concentration was fixed at 5 µM (except CP, 50 µg/mL). The correlation coefficients of DA with light irradiation time for each photosensitizer were all above 0.96 (Table 1-I and Figure 6A). Next, the correlation between concentration of each photosensitizer and DA was analyzed at a fixed irradiation time. At the 120 min reaction time, most of the correlation coefficients were higher than those between reaction time and DA, and all values were close to 1, except for RF (0.98) (Table 1-II and Figure 6B). In addition, the half-life (the time to induce 50% decolorization of MTT-F at a given concentration of photosensitizer) was calculated. The half-life value decreases as the photosensitizer concentration increases, so that the period is inversely related to the increase in concentration. The correlation coefficients between the half-life and concentration of each photosensitizer were relatively lower than those from the previous correlation; the values ranged between 0.90 and 0.95 (Table 1-III linear). A simple linear correlation model did not appear to be a good fit due to the rapid decrease in half-life with increasing photosensitizer concentration. When a logarithmic scale was applied to the half-life, a much better correlation was obtained with correlation coefficients between 0.90 and 0.99 (Table 1-III log). Since there was a negative correlation between the concentration and the half-life of MTT-F, the correlation between the photosensitizer concentrations and the reciprocal of the half-life was performed. The results showed that most of the photosensitizers had very high correlation coefficients, close to 1 (Table 1-III reciprocal and Figure 6C).

The correlation of the photosensitizing activity based on MTT-F decolorization with irradiation time, photosensitizer concentration, and MTT-F half-life was found to be highly accurate, suggesting that the photosensitizing activity could be quantified (Table 1). In Figure 6D, the DA of each photosensitizer for MTT-F is shown at 120 min irradiation time. Since each photosensitizer shows a concentration-dependent DA, the activities of different compounds must be compared at the same concentration and irradiation time. Because the DA of each photosensitizer varies with its concentration, a linear correlation of the DAs of each photosensitizer measured at three or more concentrations at the same reaction time can be analyzed and used to calculate the concentration (DC_50_) that causes a 50% decolorization of the MTT-F. Figure 6E shows the DC_50_ for MTT-F of various photosensitizers at 60 or 120 min irradiation time. The results show an order of activity with ZnPP = PPIX = ZnPc = EB ≥ MB > RF. In this method, the DC_50_ varies with irradiation time, so the reaction time must be fixed for comparing activities of different photosensitizers. Since the decolorization of MTT-F by photosensitizers occurs time-dependently, the half-life of MTT-F (the time taken for 50% decolorization of MTT-F to occur) by each photosensitizer can be calculated from the degree of decolorization measured at three or more time points. Figure 6F shows the half-lives of MTT-F by different photosensitizers at 5 µM and an order of activity with ZnPP = PPIX > ZnPc = EB = MB > RF.

This study was triggered by the previous observation that MTT response was significantly reduced in cells treated with porphyrin-based compounds without affecting cell growth. This reduction was due to the degradation of MTT-F by these photosensitizing compounds under light [10,13]. In the present study, it was confirmed that the quantitative decolorization of MTT-F occurred through direct electron transfer and a type I-like reaction with different photosensitizers, irrespective of their structural specificity, only under light conditions. Accordingly, MTT-F can be used as a sensitive and convenient probe for the quantitative analysis of various photosensitizing substances. The MTT-F method is expected to overcome the shortcomings and limitations of existing indirect methods and provide a convenient way to assess photosensitizing activity and related mechanisms.

## 3. Materials and Methods

### 3.1. Experimental Materials

MTT-F was purchased from Sigma-Aldrich Chemical Co. (Saint Louis, MO, USA). The photosensitizing agents, ZnPP, PPIX, ZnPc, RF, 5-ALA, AD, EB, and MB, used in this study were also from Sigma-Aldrich. Chlorophyll (CP, food additive grade containing corn oil; color value E10% ≥ 396) was purchased from ES Food Co. (Gunpo, Republic of Korea). MTT-F stock solution was prepared in DMSO at a concentration of 20 mM. Stock solutions for ZnPP, PPIX, 5-ALA, AD, EB, and MB were also prepared in DMSO at 20 mM concentration. RF, ZnPc, and CP were dissolved in DMSO at concentrations 10, 0.5 mM, and 100 mg/mL, respectively. All stock solutions were stored at −80 °C or −20 °C. All other chemicals were purchased from Sigma-Aldrich Chemical Co.

### 3.2. Fluorescent Lamp Lighting System

A light source used in this study was a fluorescent lamp commonly used in the home (27 W, FPL-27W EX-D, Philips Korea, Seoul, Republic of Korea) and had more than 3 emission peaks around 440, 550, and 620 nm (Figure S1A). Illuminance and irradiance at different irradiation distance were analyzed using a light meter (Traceable 62344-944, VWR, Radnor, PA, USA) and an irradiance meter (IRR1-Sol, Fluke, Everett, WA, USA), respectively.

### 3.3. Analysis of MTT-F Decolorization by Photosensitizers under Light Irradiation

MTT-F (400 μM, 50 μL) dissolved in DMSO was mixed with 50 μL of each photosensitizing agent (5 μM each or 50 μg/mL for CP) diluted in DMSO on a 96-well plate. The mixture solution was irradiated using a household fluorescent lamp (27 W, FPL-27W EX-D) with illuminance of ~2000 lx for 24 h at room temperature. Changes in absorbance of MTT-F at each time point were measured using a microplate reader (Spectra Max M2, Molecular device, Sunnyvale, CA, USA) at 550 nm. In order to analyze illuminance-dependent effect on MTT-F decolorization by each photosensitizer, the fluorescent lamp was set to the irradiation distance of 35 cm (~2000 lx), 52 cm (~1000 lx), and 66 cm (~500 lx) (Figure 1B).

### 3.4. Characterization of MTT-F Decolorization by Different Radicals and in a Vacuum Condition

Effects of different radicals on the decolorization of MTT-F were explored using ABTS, DPPH, and AAPH radicals. ABTS radical was generated by incubating in the dark at 25 °C for 24 h after mixing 10 mM ABTS and 10 mM potassium persulfate at a ratio of 7.4:2.6 (*v*/*v*). The ABTS radical solution was diluted to an appropriate absorbance of 0.7–0.8. Different concentrations of MTT-F (50 μL) were mixed with 150 μL of the ABTS solution. The absorbance spectra and peak absorbance of ABTS radical and MTT-F at 734 nm and 550 nm, respectively, were analyzed immediately (Spectra Max M2). DPPH radical in MeOH (60 μL) was mixed with equal volume of MTT-F dissolved in DMSO, and absorbance changes at 517 and 550 nm were monitored after 30 min of reaction in the dark. Different concentrations of AAPH (0–20 mM, 50 μL) were mixed with 50 μL of MTT-F (400 μM), and changes in color intensity of MTT-F were also measured at 550 nm in a dark place at 25 °C during 24 h. In addition, in order to check the reactivity of MTT-F with ROS, absorbance changes at 550 nm of a reaction mixture of MTT-F (400 μM) with H_2_O_2_ (0–800 μM) at a volume ratio of 1:1 were analyzed in a dark place at 25 °C during 6 h (Spectra Max M2).

### 3.5. Evaluation of MTT-F Decolorization by Photosensitizers in a Vacuum Condition

Decolorization of MTT-F by photosensitizers was also compared under both normal aeration and vacuum conditions. The reaction mixture of each photosensitizer and MTT-F was placed in a clear glass flask, and a vacuum pump (Rocker 300, Rocker Scientific, Kaohsiung City, Taiwan) was used to maintain a vacuum condition (~650 mmHg) in the flask, while the fluorescent light was irradiated for 15 min. Decolorization of MTT-F by photosensitizers was monitored at 550 nm immediately after the reaction. The color changes in MTT-F were also compared under the same conditions without light irradiation or vacuum application.

### 3.6. Analysis of MTT-F Decolorization Product Using LC-MS/MS and Its Reduction by Cells

For the analysis and identification of the decolorized products from MTT-F, ultra-performance liquid chromatography electrospray ionization mass spectrometry (UPLC-ESI-MS) was applied (UPLC-MS/MS, ACQUITY UPLC and SYNAPT G2, Waters, Milford, MA, USA). The sample was prepared to be ~50% decolorized by reacting MTT-F with ZnPP or PPIX using the method described above. The C18 packed column (2.1 × 100 mm, 1.6 μm particle size) was used, and the temperature was set to 40 °C. As a mobile phase, 0.1% formic acid in water (A) and 0.1% formic acid in acetonitrile (B) were used. Gradients were given to 0–3 min: A 95%, 3–25 min: A 80%, and 25–26 min: A 1%, and 26–27 min: A 95% and 27–30 min: A 95% were initialized. The sample was analyzed at a rate of 0.2 mL/min by injecting 4 μL. MS analysis was performed under the conditions of a voltage of 3.0 kV, desolvation temperature of 450 °C, and gas flow rate of 800 L/h. The detailed analytical conditions are summarized in Table S1.

To confirm if the decolorized MTT-F was reduced to its original formazan form by living cells, more than 90% decolorized MTT-F was prepared by incubating of fresh MTT-F (2.5 mM) with PPIX (50 µM) under light. HaCaT cells were treated with decolorized MTT-F diluted 10 times with culture media, and then further incubated for 2 h and lysed using DMSO. MTT-F color intensity was analyzed at 550 nm before cell treatment, immediately after cell lysis, and after 2 h of incubation under light irradiation. Absorbance spectra of fresh MTT-F and those decolorized by PPIX, as well as those reduced by cells from the decolorized formazan, were also compared.

### 3.7. Data Analysis

Each experiment was repeated by more than 3 times, and all values represent the mean ± standard deviation. Statistical significance was evaluated by Student’s *t*-test. One-way ANOVA with Tukey’s HSD (honestly significant difference) test was used for comparing multiple results using the SPSS software program (IBM SPSS Statistics 24, SPSS Inc., Chicago, IL, USA).

## 4. Conclusions

There is a growing interest in photosensitizers that have various effects in food, medicine, cosmetics, etc., under light. However, analytical studies of photosensitizing activity are very limited, and quantifying this activity is challenging. Since previous studies indicated that MTT-F was decolorized by certain photoreactive compounds such as porphyrins under light, the method for analyzing photosensitizing activity of various types of photosensitizers was developed using MTT-F as a probe. The seven photosensitizers used in this study induced quantitative decolorization of MTT-F as a function of light exposure time, light intensity, and photosensitizer concentration. MTT-F decolorization is preferentially caused by the type I-like reaction that reacts directly with light-activated photosensitizers, rather than with ROS or secondary oxidation products generated from photosensitizing reaction. The oxidized and decolorized MTT-F exhibited a base peak of −1 *m*/*z* compared to MTT-F and could be reversibly reduced to its original formazan form by living cells. The response of MTT-F decolorization by photosensitizers was highly correlated to their photosensitizing activity of the reaction factors including the exposure time to light, the half-life of MTT-F color, and the concentrations of the photosensitizers. The present results suggest that MTT-F can be a reliable probe for the quantitative analysis of photosensitizing activity. In conclusion, this method is expected to contribute to the prediction of effects and prevention of problems caused by photosensitizers in the food, pharmaceutical, and cosmetic industries, as well as provide a source technology for the discovery and screening of photosensitizers needed in various academic and industrial fields.

## Figures and Tables

**Table 1:** Linear correlation coefficients between photosensitizing activities and irradiation time (A-I) or photosensitizer concentrations (A-II) and between photosensitizer concentrations and half-lives of MTT-F (A-III).

		ZnPP	PPIX	ZnPc	RF	EB	MB	CP
Range	2000 lx	0–5 μM 0–240 min	0–5 μM 0–240 min	0–5 μM 0–240 min	0–10 μM 0–480 min	0–5 μM 0–240 min	0–5 μM 0–240 min	0–50 μg/mL 0–240 min
I. Irradiation time vs. Activity								
	R^2^	0.9818	0.9906	0.9768	0.9601	0.9952	0.9994	0.9921
II. Concentration vs. Activity (120 min)								
	R^2^	0.9939	0.9963	0.9955	0.9846	0.9989	0.9996	0.9997
III. Concentration vs. Half-life								
Linear scale	R^2^	−0.9046	−0.9161	−0.9224	−0.8973	−0.9063	−0.9468	−0.9151
Log scale	R^2^	−0.9689	−0.9754	−0.9788	−0.9646	−0.9699	−0.9905	−0.9046
Reciprocal	R^2^	0.9948	0.9992	1.0000	0.9756	0.9985	1.0000	0.9980

## Data Availability

Data are available from the corresponding author upon reasonable request.

## Supplementary Materials

The following supporting information can be downloaded at: https://www.mdpi.com/article/10.3390/molecules29112471/s1.
