# Supplementary material for: Development of a Convenient and Quantitative Method for Evaluating Photosensitizing Activity Using Thiazolyl Blue Formazan Dye
Source: Molecules. 2024 May 24;29(11):2471. doi: 10.3390/molecules29112471 (PMC11173384; doi:10.3390/molecules29112471)
Supplement: Supplementary file 1 [file molecules-29-02471-s001.zip › molecules-3007316-supplementary.pdf]

Supplementary Figure S1. Characteristics of the fluorescent light used in the current study. Emission spectrum (A). Photostability of MTT-F was also analyzed under the irradiation of the fluorescence light (2000 lx) for 24 h (B).

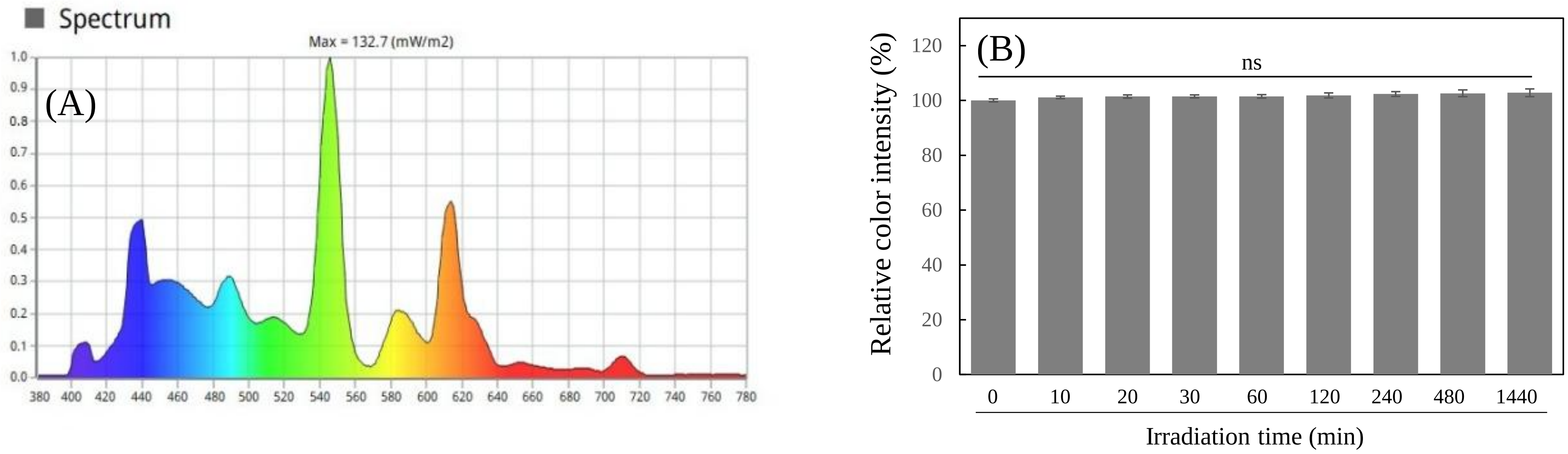

Supplementary Figure S1A and B

Supplementary Figure S2. Analysis of photosensitizing properties of different photosensitizers using DCFH. HCT 116 cells were incubated with DCFH-DA (2',7'-Dichlorofluorescein diacetate) (20 μM). After 2 h, the cells were lysed with DMSO, and the cell lysates containing DCFH mixed with different concentrations of ZnPP and ZnPc (A), PPIX and RF (B), and 5-ALA and AD (C), (1,25, 2.5, and 5 μM each) were incubated under the fluorescence light (2000 lx) or in the dark with 5 μM of different photosensitizers (D). After 120 min incubation, DCF fluorescence were analyzed at an emission 535 nm, excitation 485 nm. Each value represents the mean±SD (n=4). Different letters indicate a significant difference ($p<0.05$) based on one-way ANOVA and the Tukey's HSD test. ns; not significant.

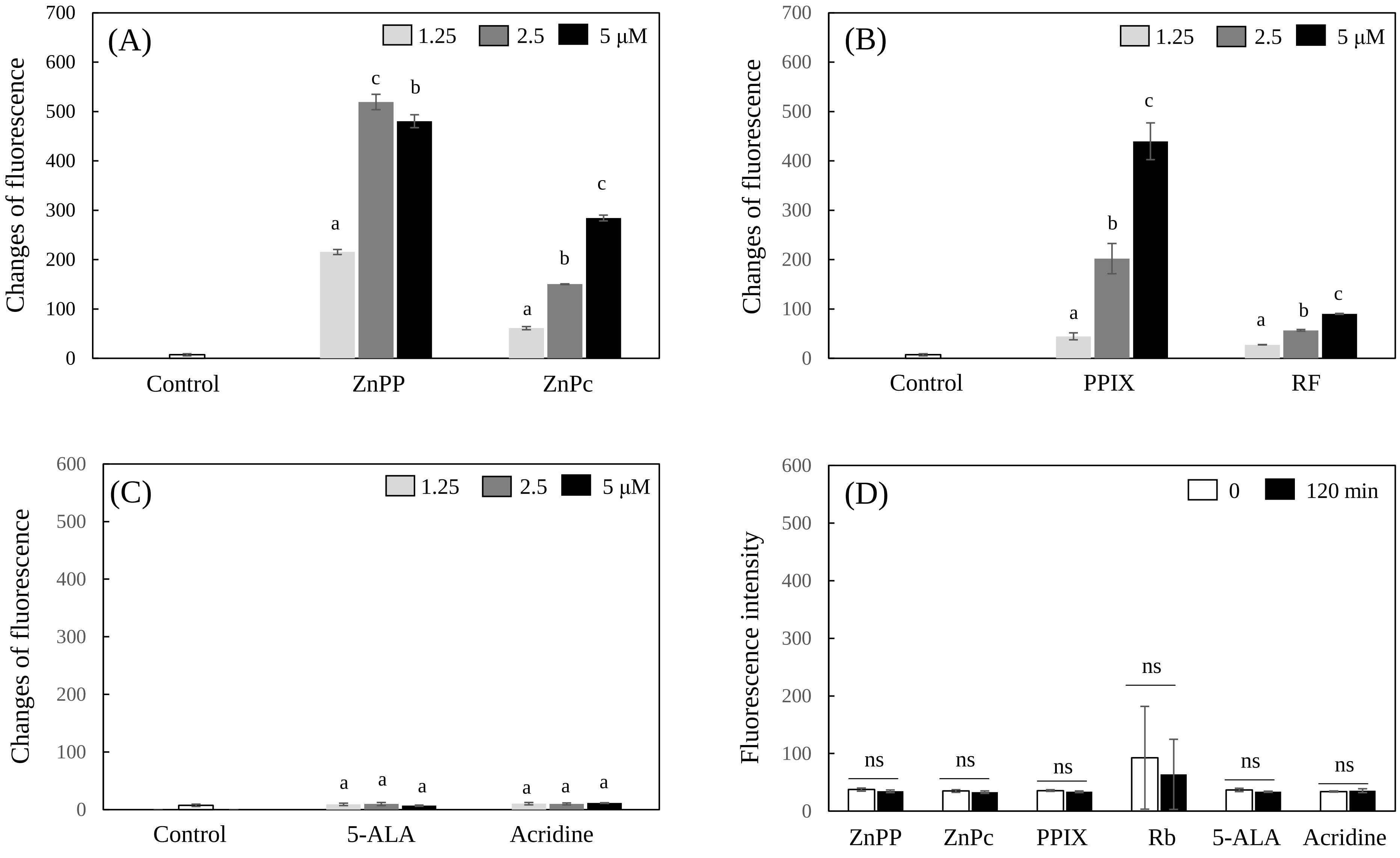

Supplementary Figure S2A-D

Supplementary Figure S3 Analysis of photosensitizing properties of different photosensitizers using TBA. Linoleic acid (0.5%) mixed with different concentrations of photosensitizers (each 0-10 μM or 0-100 μg/mL for CP) in 0.4% sodium dodecyl sulfate (SDS) was irradiated under the fluorescence light (4000 lx) for 2 h. Thiobarbituric acid reactive substance (TBARS) in the reaction mixture (120 μL) was produced by heating at 95 °C for 10 mint after adding 60 μL of 1 M trichloroacetic acid, 60 μL of 0.8% TBA, and 10 μL of 5% butylated hydroxytoluene diluted in ethanol, and the formation of TBARS was analyzed at 532 nm. Each value represents the mean±SD (n=4). Different letters indicate a significant difference (p<0.05) based on one-way ANOVA and the Tukey's HSD test.

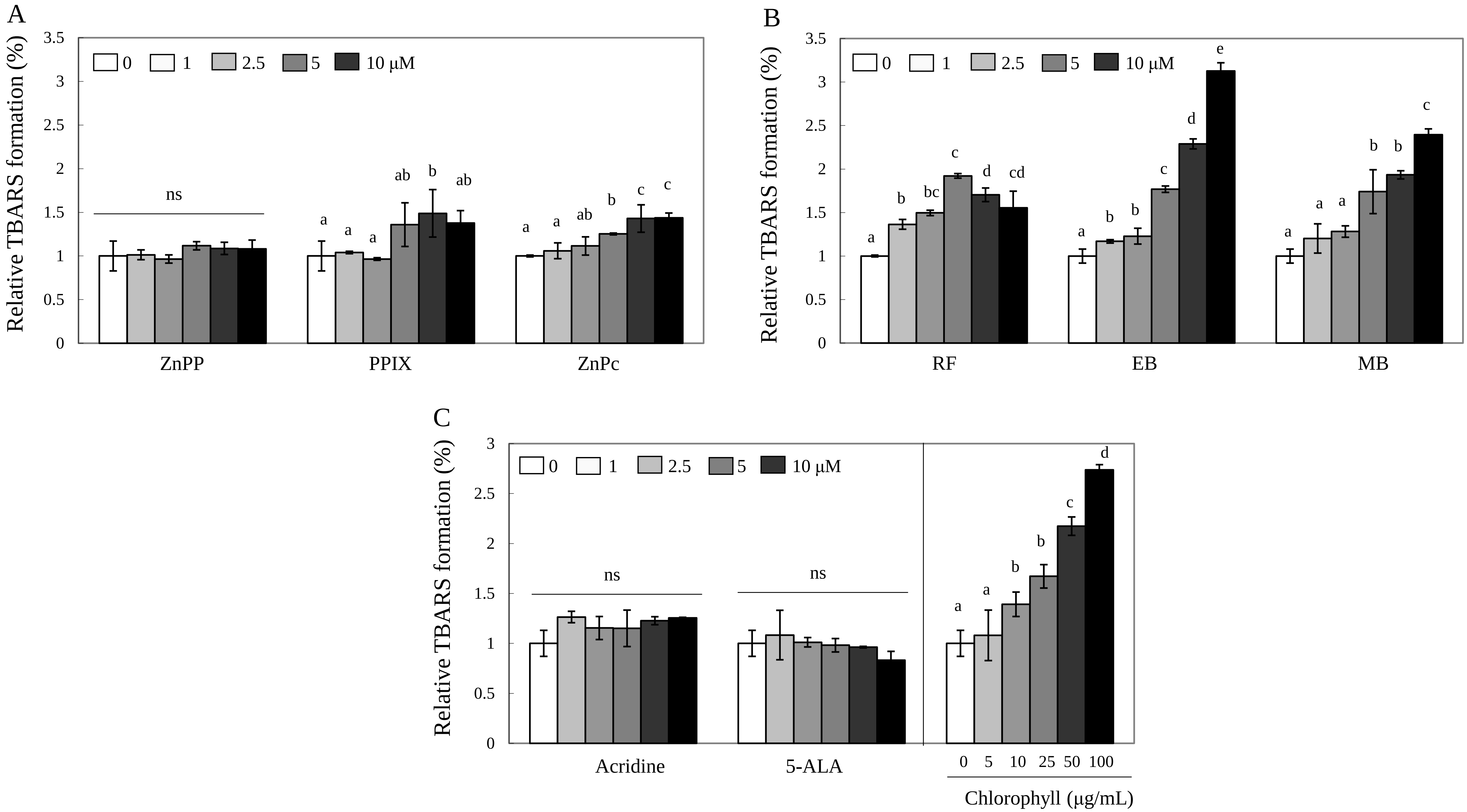

Supplementary Figure S3A-C

Supplementary Figure S4. The chromatograms of the reaction products from MTT-F with photosensitizers and MS/MS spectra of the decolorized MTT-F. The reaction was performed as described in Fig. 5. The chromatograms of the reaction products from MTT-F incubated with ZnPP (A) and PPIX (B) under light were shown. MS/MS spectra of the decolorized MTT-F (peak 2) after reaction with ZnPP (C) and PPIX (D) were also shown.

(A) MTT-F with ZnPP

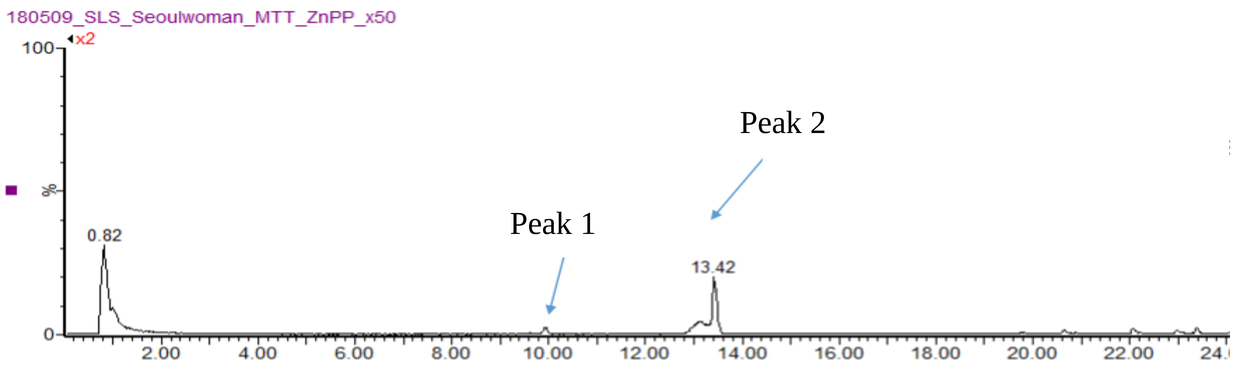

(B) MTT-F with PPIX

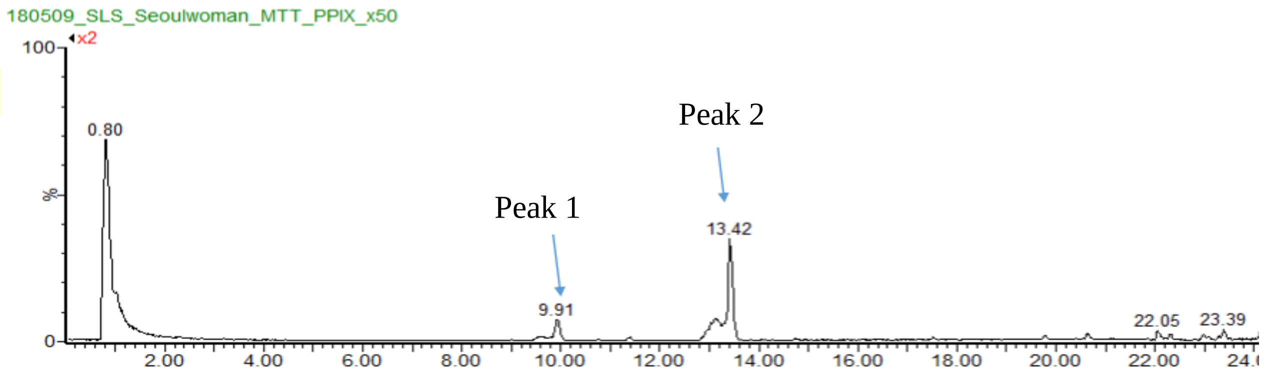

(C) MS/MS of decolorized MTT-F by ZnPP (Peak 2)

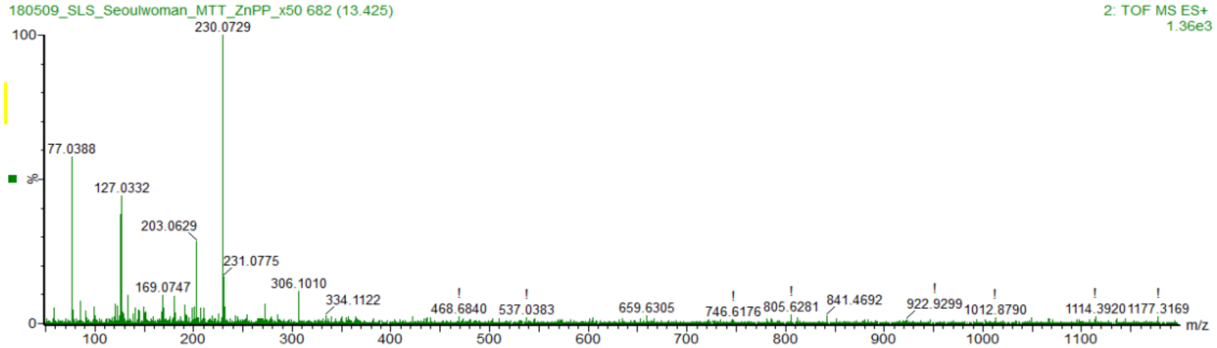

(D) MS/MS of decolorized MTT-F by PPIX (Peak 2)

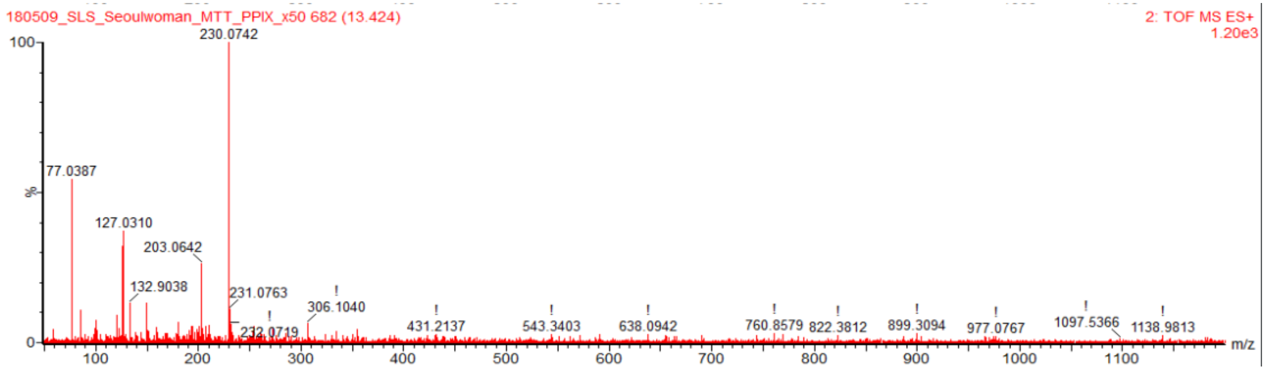

Supplementary Figure S4A-D

Supplementary Table S1. Conditions for analyzing reaction products of MTT-F with photosensitizers using the UPLC-ESI-MS system

LC Condition (Waters® ACQUITY™ UPLC)			
Column	CORTECS™ UPLC® C18 1.6 μm (2.1x100 mm)		
	Temperature : 40 ℃		
Mobile phase	A : 0.1% FA in DW		
	B : 0.1% FA in ACN		
Gradient	Time (min)	A (%)	B (%)
	0	5	95
	3	5	95
	25	20	80
	26	99	1
	27	5	95
	30	5	95
Flow rate	0.2 ml/min		
Injection volume	4 μl		
**MS Condition (SYNAPT™ G2)**			
Ionization Mode	ESI+		
Capillary	3.0 kv		
Sampling cone	35 V		
Extraction cone	4.0		
Source	120 ℃		
Desolvation	450 ℃		
Cone gas	100 L/h		
Desolvation gas	800 L/h		
